# Unravelling the interfacial interaction in mesoporous SiO_2_@nickel phyllosilicate/TiO_2_ core–shell nanostructures for photocatalytic activity

**DOI:** 10.3762/bjnano.11.165

**Published:** 2020-12-09

**Authors:** Bridget K Mutuma, Xiluva Mathebula, Isaac Nongwe, Bonakele P Mtolo, Boitumelo J Matsoso, Rudolph Erasmus, Zikhona Tetana, Neil J Coville

**Affiliations:** 1 DSI-NRF Centre of Excellence in Strong Materials and Molecular Sciences Institute, School of Chemistry, University of the Witwatersrand, WITS 2050, Johannesburg, South Africa; 2 Department of Natural Resources and Materials, Botswana Institute for Technology Research and Innovation, 50654 Machel Drive, Gaborone, Botswana; 3 School of Physics, University of Witwatersrand, WITS 2050, Johannesburg, South Africa

**Keywords:** bandgap energy, core–shell, dye degradation, nickel phyllosilicate, photocatalysts

## Abstract

Core–shell based nanostructures are attractive candidates for photocatalysis owing to their tunable physicochemical properties, their interfacial contact effects, and their efficacy in charge-carrier separation. This study reports, for the first time, on the synthesis of mesoporous silica@nickel phyllosilicate/titania (mSiO_2_@NiPS/TiO_2_) core–shell nanostructures. The TEM results showed that the mSiO_2_@NiPS composite has a core–shell nanostructure with a unique flake-like shell morphology. XPS analysis revealed the successful formation of 1:1 nickel phyllosilicate on the SiO_2_ surface. The addition of TiO_2_ to the mSiO_2_@NiPS yielded the mSiO_2_@NiPS/TiO_2_ composite. The bandgap energy of mSiO_2_@NiPS and of mSiO_2_@NiPS/TiO_2_ were estimated to be 2.05 and 2.68 eV, respectively, indicating the role of titania in tuning the optoelectronic properties of the SiO_2_@nickel phyllosilicate. As a proof of concept, the core–shell nanostructures were used as photocatalysts for the degradation of methyl violet dye and the degradation efficiencies were found to be 72% and 99% for the mSiO_2_@NiPS and the mSiO_2_@NiPS/TiO_2_ nanostructures, respectively. Furthermore, a recyclability test revealed good stability and recyclability of the mSiO_2_@NiPS/TiO_2_ photocatalyst with a degradation efficacy of 93% after three cycles. The porous flake-like morphology of the nickel phyllosilicate acted as a suitable support for the TiO_2_ nanoparticles. Further, a coating of TiO_2_ on the mSiO_2_@NiPS surface greatly affected the surface features and optoelectronic properties of the core–shell nanostructure and yielded superior photocatalytic properties.

## Introduction

Textile dyes and organic compounds are major water pollutants, which create an environmental hazard to aquatic systems and humanity. For instance, textile dyes of the methylene family, such as methylene blue (MB), methyl orange (MO), and methyl violet (MV), have detrimental toxicological and ecological effects on human life and the environment [1–2]. Thus, considerable efforts have been garnered towards finding efficient, reliable, and eco-friendly water-treatment and decontamination techniques in order to mitigate this issue [3–4]. Among the various techniques, the use of semiconducting photocatalysts for light-stimulated degradation of dye pollutants has been extensively investigated [5]. Owing to its chemical inertness, low cost, and non-toxicity, titanium dioxide (TiO_2_) has been widely used as a photocatalyst in the degradation of dyes in textile industries as well as in water-treatment systems [5–6]. There are three different phases of TiO_2_, namely anatase, rutile, and brookite. Compared to the rutile and brookite phases, the anatase phase has been extensively used for photocatalysis owing to its enhanced surface properties [7–10].

In a typical photocatalytic process, photons of energy greater than the bandgap energy of TiO_2_ excite electrons to the conduction band leaving holes in the valence band. The photoexcited electrons and the presence of holes result in the oxidization of organic dyes via a free-radical mechanism. However, TiO_2_ is a wide-bandgap semiconductor (3.0–3.3 eV), which can only absorb UV light and it easily undergoes electron–hole recombination [11]. To circumvent this problem, the electron–hole recombination can be inhibited by loading metals, such as Ni [12], V, Fe [13], Ag [14], and Cu–Ni [15], on the TiO_2_ surface, which accelerates the formation of hydroxyl radicals and, consequently, improves the photocatalytic activity of TiO_2_. In contrast, the doping of TiO_2_ with metal oxides, such as ZrO_2_ [16] and SiO_2_ [17], influence the morphology and surface features of the resulting binary metal oxide semiconductors. Moreover, these binary metal oxide semiconductors act as charge-transfer catalysts and significantly reduce the electron–hole recombination [18–19].

Another factor that affects the photocatalytic activity of TiO_2_, is its adsorption capacity for dye molecules. The adsorption capacity of TiO_2_ can be readily improved by modifying its surface charge density or by increasing its surface area and pore volume [5,20–21]. Further, SiO_2_ is a good adsorptive material that facilitates easy adsorption of organic molecules and their transfer onto the active sites of TiO_2_ [22–23]. Additionally, the interaction between SiO_2_ and TiO_2_ could result in the creation of oxygen vacancies that promote charge-transfer processes and, hence, enhance the photocatalytic activity [24–25]. One method to maximize the SiO_2_–TiO_2_ interaction is via the synthesis of core–shell nanostructures or nanocomposites [18–19,26–27]. Ikeda et al. [26] reported an improved photodecomposition of acetic acid by using a titania core@hollow silica shell nanostructured catalyst. Similarly, Ren et al. [27] observed an improved photocatalytic degradation of rhodamine blue dye in the presence of rattle-type TiO_2_@void@SiO_2_ nanostructures. They attributed the improved photoactivity to the ease of dye molecules to access the TiO_2_ active sites through the void space between the core and the shell. Although rattle-type TiO_2_@void@SiO_2_ systems could provide high dye adsorption ability, a core–shell architecture (TiO_2_@SiO_2_) provides a better separation rate of the photogenerated electrons and holes by restricting the electron–hole recombination through the close interaction of silica with the titania surface [23]. Thus, silica-based core–shell nanocomposites offer added advantages of manipulating the pore structure, surface area, morphology, and catalyst reactivity [28].

Unlike the metal oxide–metal oxide composites, the use of metal oxide core@metal nanocomposites as dopants for titania photocatalysts is rarely reported. Nickel-based nanomaterials are of great interest for photocatalytic activity owing to their low cost, high optical absorption coefficients, and low bandgap energies [29–31]. Most importantly, the formation of a Schottky barrier between nickel species and titania can restrict the electron–hole recombination [29]. Thus, a system that seeks to combine a porous silica core as a good support for nickel-based nanomaterials with titania nanoparticles could provide a synergistic effect for good photocatalytic activity. Nickel phyllosilicate structures can be readily generated by the reaction of nickel species with silica [32]. Generally, there are two phases of nickel phyllosilicate that can be achieved, that is, a 2:1 phyllosilicate with the structural formula Ni_3_Si_4_O_10_(OH)_2_ and a 1:1 phyllosilicate with the structural formula Ni_3_Si_2_O_5_(OH)_4_ [33]. While 1:1 nickel phyllosilicate can be obtained either by a hydrothermal method or a deposition-precipitation method, the 2:1 nickel phyllosilicate is only formed under hydrothermal conditions [32–34].

Recently, several researchers have reported on the generation of nickel phyllosilicate catalysts for hydrogenation reactions, methane reforming, and hydrogen evolution [35–40]. Wang et al. [38] reported the growth of nickel phyllosilicate by simultaneous reaction of a silica precursor (tetraethylorthosilicate), nickel chloride, water, and urea in a hydrothermal reactor at 210 °C for 12 h. They obtained NiPS with a sheet-like morphology, which was then used as a catalyst for the hydrogenation of styrene. More recently, Ghiat et al. [39] reported on the photocatalytic properties of nickel phyllosilicates for hydrogen production. Their nickel phyllosilicate, displaying a surface area of 95 m^2^·g^−1^, was obtained via a hydrothermal treatment method using solid SiO_2_ spheres, urea, and nickel nitrate hexahydrate.

Although NiPS compounds are extensively used as catalysts, to our knowledge, reports on the use of core–shell based nickel–phyllosilicate composites in dye photodegradation are yet to be reported. Therefore, in this study we report on the growth of mesoporous SiO_2_@NiPS and titania-coated mSiO_2_@NiPS (mSiO_2_@NiPS/TiO_2_) core–shell nanostructures using a simple deposition-precipitation method. Transmission electron microscopy (TEM), N_2_ physisorption analysis, diffuse reflectance UV–visible spectroscopy, and X-ray photoelectron spectroscopy (XPS) were used to elucidate the morphological and textural features, optoelectronic properties, and elemental composition of the core–shell nanomaterials. These core–shell nanostructures were also employed as catalysts in a model reaction, that is, the photodegradation of methyl violet dye. For comparison, the photocatalytic properties of pristine TiO_2_ were also investigated. It is postulated that the controlled surface area of the mesoporous SiO_2_ as well as the formation of a core–shell network with a flake-like NiPS structure on the surface aided in creating a good support for the TiO_2_ nanoparticles. Further, a coating of TiO_2_ on the SiO_2_@NiPS surface greatly impacted the surface features and optoelectronic properties of the core–shell nanostructure and yielded superior photocatalytic properties.

## Results and Discussion

### Morphological features, textural, and optical properties

**Figure 1:**
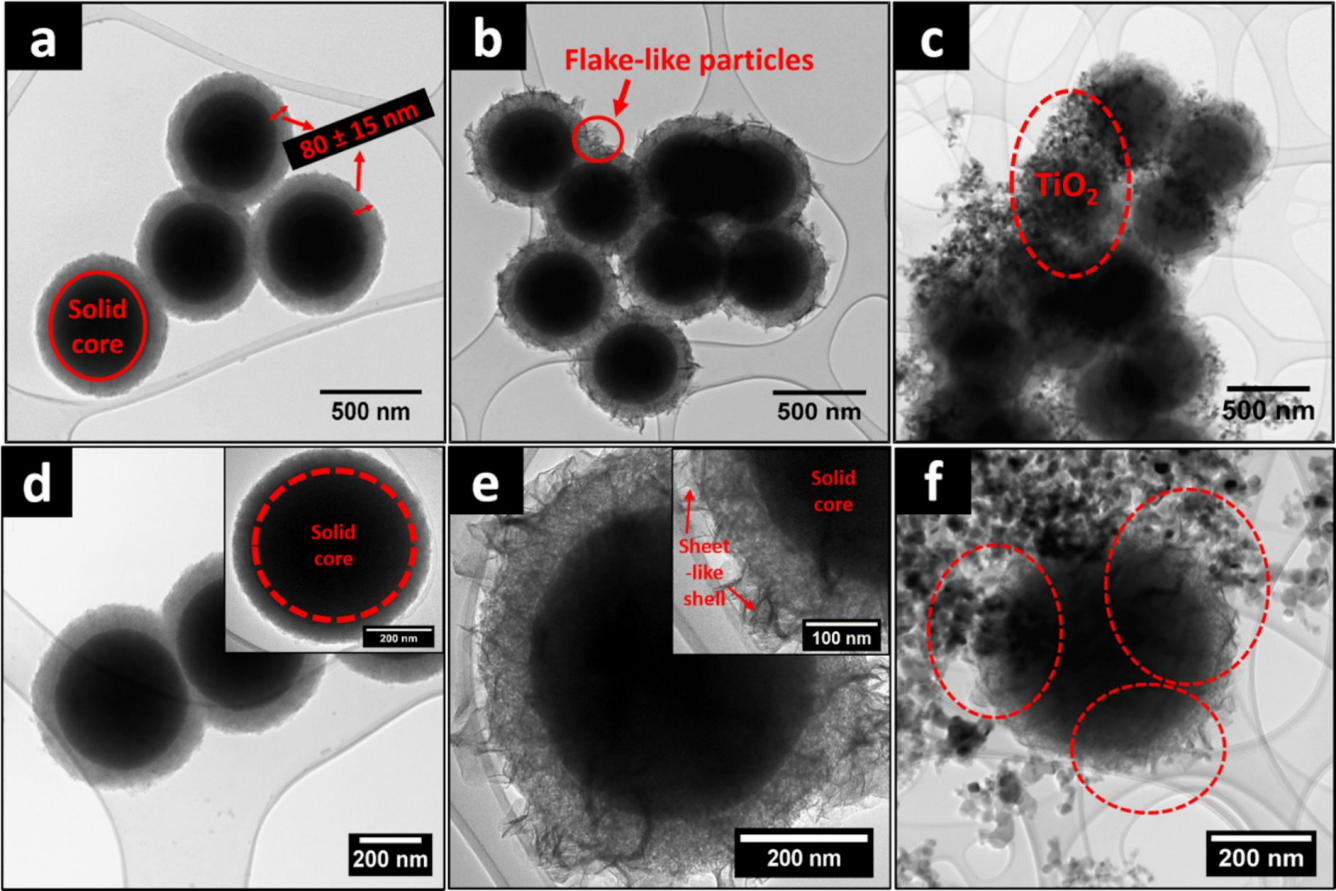
TEM images of (a, d) mesoporous SiO_2_, (b, e) mSiO_2_@NiPS, and (c, f) mSiO_2_@NiPS/TiO_2_.

The three-step synthesis involved the generation of the SiO_2_ spheres, the reaction of the surface of SiO_2_ with Ni to give a NiPS-covered SiO_2_ spheres, and the addition of TiO_2_ to create the final product. SEM images show the spherical morphology of the mSiO_2_ and mSiO_2_@NiPS nanostructures (Supporting Information File 1, Figure S1a,b). The mSiO_2_@NiPS/TiO_2_ composite comprises spherical mSiO_2_@NiPS with aggregates of TiO_2_ on the surface (Supporting Information File 1, Figure S1c). The TEM images of the mesoporous SiO_2_ spheres show more clearly that the sample comprises an inner solid core and an outer mesoporous shell (Figure 1a and Figure 1d). The inner diameter of the SiO_2_ spheres was measured to be 415 ± 11 nm with a shell thickness of 80 ± 15 nm.

After addition of Ni salt and thermal treatment, the core–shell nanospheres have been maintained but they are now covered with flake-like structures on the surface (Figure 1b). The shell thickness increased to 92 ± 12 nm, implying the successful formation of nickel phyllosilicate on the silica surface (mSiO_2_@NiPS). The high-magnification TEM images of the samples show sheet-like structures on the SiO_2_ shell, further confirming the formation of NiPS on the SiO_2_ surface (Figure 1e). A similar flake-like structure on the surface of silica was observed by Guo et al. [41] on mesoporous SiO_2_/Ni_3_Si_2_O_5_(OH)_4_ core–shell microspheres. Ni_3_Si_2_O_5_(OH)_4_ corresponding to 1:1 nickel phyllosilicate comprises a brucitic sheet of Ni(II) cations that are octahedrally coordinated and connected to an interlinked SiO_4_ tetrahedra layer. This was further confirmed by XRD data. Figure S2a in Supporting Information File 1 shows the XRD patterns for mesoporous SiO_2_ and mSiO_2_@NiPS. A broad peak was observed at 2θ = 22° in the SiO_2_ and mSiO_2_@NiPS samples, corresponding to the presence of amorphous silica. In the mSiO_2_@NiPS sample, this peak could be correlated to an overlap of silica with the (002) plane of nickel phyllosilicate [35]. Additional peaks at 2θ = 34°, 36°, and 60° in the mSiO_2_@NiPS sample were assigned to (200), (202), and (060) diffractions of the 1:1 NiPS nickel phyllosilicate structure Si_2_Ni_3_O_5_(OH)_4_ [42–43].

Basically, NiPS is formed upon the precipitation of nickel species onto a silica surface after basification of a nickel(II) solution [44]. Depending on the deposition-precipitation time, the molar ratio between urea and nickel precursor, and the silica surface area, either nickel hydroxide or nickel phyllosilicate can be obtained [34]. Nickel hydroxide is formed when precipitation takes place due to supersaturation [32]. In contrast, precipitation of nickel phyllosilicate occurs only due to the interaction between the nickel(II) species and silica after the homogeneous addition of hydroxide ions throughout the whole solution. Therefore, the formation of nickel phyllosilicate in this study could be postulated as follows: Dispersing the silica spheres in distilled water yielded a white silica solution. After the addition of urea, at 90 °C, urea hydrolysis took place reducing the nickel precursor to nickel species, which diffused into the silica layers through the mesoporous shell. The nickel species reacted with the surface hydroxides to give nickel phyllosilicate via a Ni–O–Si polymerization reaction.

TiO_2_ (*d*_TiO2_ = 30 ± 9 nm) was then added to the SiO_2_@NiPS material. The coverage of the spherical mSiO_2_@NiPS by aggregates of TiO_2_ nanoparticles to give mSiO_2_@NiPS/TiO_2_ can be clearly seen (Figure 1c and Figure 1f). EDX spectra confirmed the presence of nickel and silica in the mSiO_2_@NiPS, as well as that of nickel, titania, and silica in mSiO_2_@NiPS/TiO_2_ (Supporting Information File 1, Figure S3).

**Figure 2:**
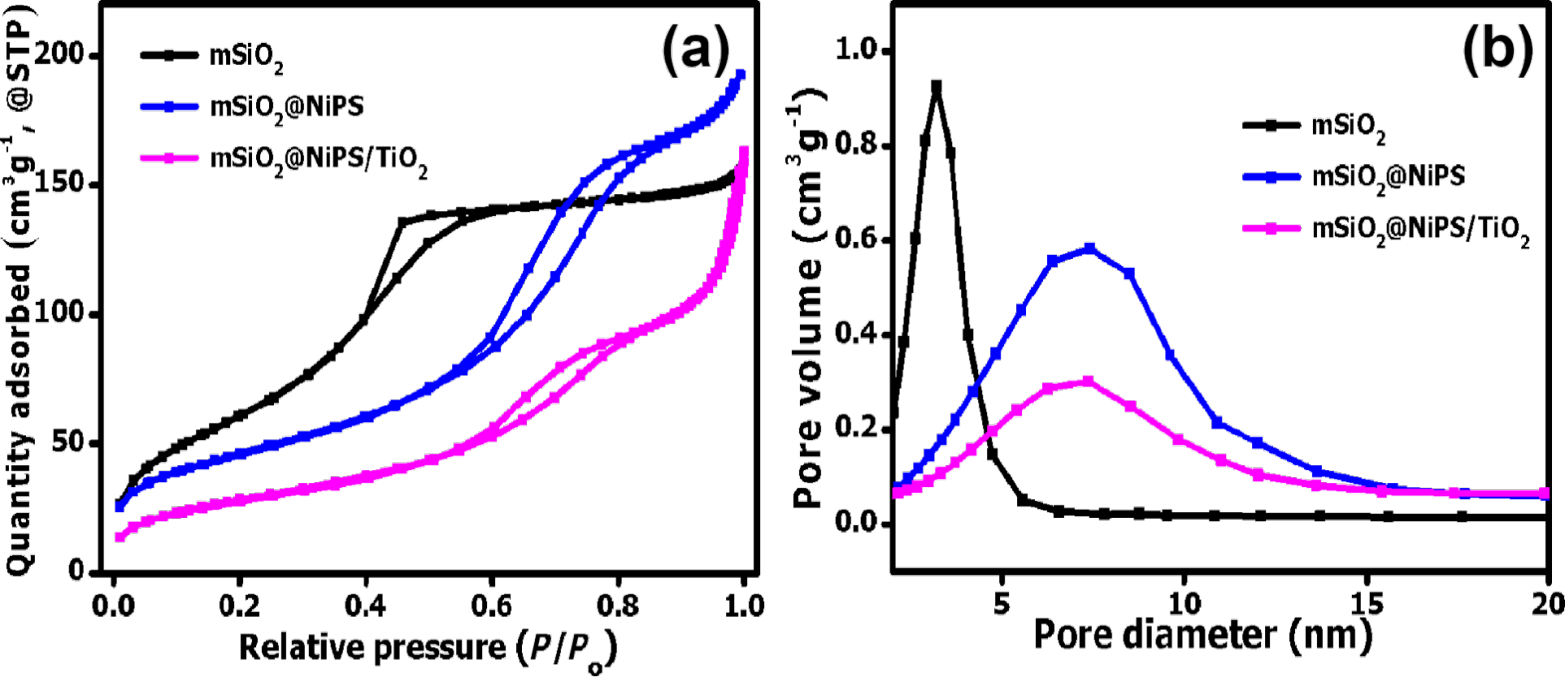
(a) N_2_ adsorption–desorption isotherms and (b) pore size distribution of mSiO_2_, mSiO_2_@NiPS and mSiO_2_@NiPS/TiO_2_.

The textural properties of the core–shell nanostructures were evaluated using N_2_ physisorption analysis. Figure 2a shows that the nitrogen gas adsorption–desorption isotherms of mSiO_2_ and the mSiO_2_@NiPS core–shell nanostructure are of type IV, demonstrating the presence of a mesoporous structure associated with capillary condensation [45–46]. The mSiO_2_ spheres exhibited a type-H2 hysteresis loop confined at 0.35 < *P*/*P*_0_ < 0.6, indicating the presence of randomly interconnected pore systems [47]. In contrast, the mSiO_2_@NiPS core–shell nanostructures showed a type-H1 hysteresis loop, implying the existence of agglomerates with cylindrical pores [45,48]. Similar to the mSiO_2_@NiPS composite, the mSiO_2_@NiPS/TiO_2_ exhibited a type-IV isotherm with a type-H1 hysteresis loop, illustrating the presence of mesopores with randomly agglomerated pores [49]. TiO_2_, on the other hand, displayed a type-II isotherm characteristic of a nonporous material (Supporting Information File 1, Figure S2b).

The pore size distribution in the SiO_2_ was narrow (2–5 nm) and in the mesoporous range with an average pore size of 3.2 nm (Figure 2b). After formation of the NiPS, the average pore size increased to 6.3 nm in the mSiO_2_@NiPS spheres (Table 1). This can be attributed to the formation of the sheet-like NiPS on the SiO_2_ surface and/or a possible partial etching of silica during the deposition-precipitation process by the alkaline solution (urea/ammonia) [45–46,49]. In addition, the pore size distribution of the mSiO_2_@NiPS core–shell nanocomposite was broader (2–15 nm) than that of mSiO_2_ (Figure 2b). During the formation of the mSiO_2_@NiPS/TiO_2_ composite, the pore diameter slightly increased to 7.7 nm, indicating the presence of interparticle voids resulting from the presence of TiO_2_ agglomerates. Table 1 shows BET surface area, pore volume, and pore diameter of the mSiO_2_, mSiO_2_@NiPS, TiO_2_, and SiO_2_@NiPS/TiO_2_ samples. The surface area of mesoporous SiO_2_ and mSiO_2_@NiPS was 231 m^2^/g and 164 m^2^/g, respectively, indicating the removal of the porous silica surface and the successful growth of sheet-like NiPS particles on top of the SiO_2_ matrix. The addition of TiO_2_ (51 m^2^/g) as expected, led to a lower surface area of the mSiO_2_@NiPS/TiO_2_ composite (103 m^2^/g) compared to the mSiO_2_@NiPS core–shell nanostructures.

**Table 1:** Surface area data of the mSiO_2_, mSiO_2_@NiPS, and mSiO_2_@NiPS/TiO_2_ samples.

Material	Surface area (m^2^/g)	Pore diameter (nm)

mSiO_2_	235.8 ± 5.8	3.2
mSiO_2_@NiPS	164.6 ± 0.9	6.3
mSiO_2_@NiPS/TiO_2_	102.7 ± 0.9	7.7

**Figure 3:**
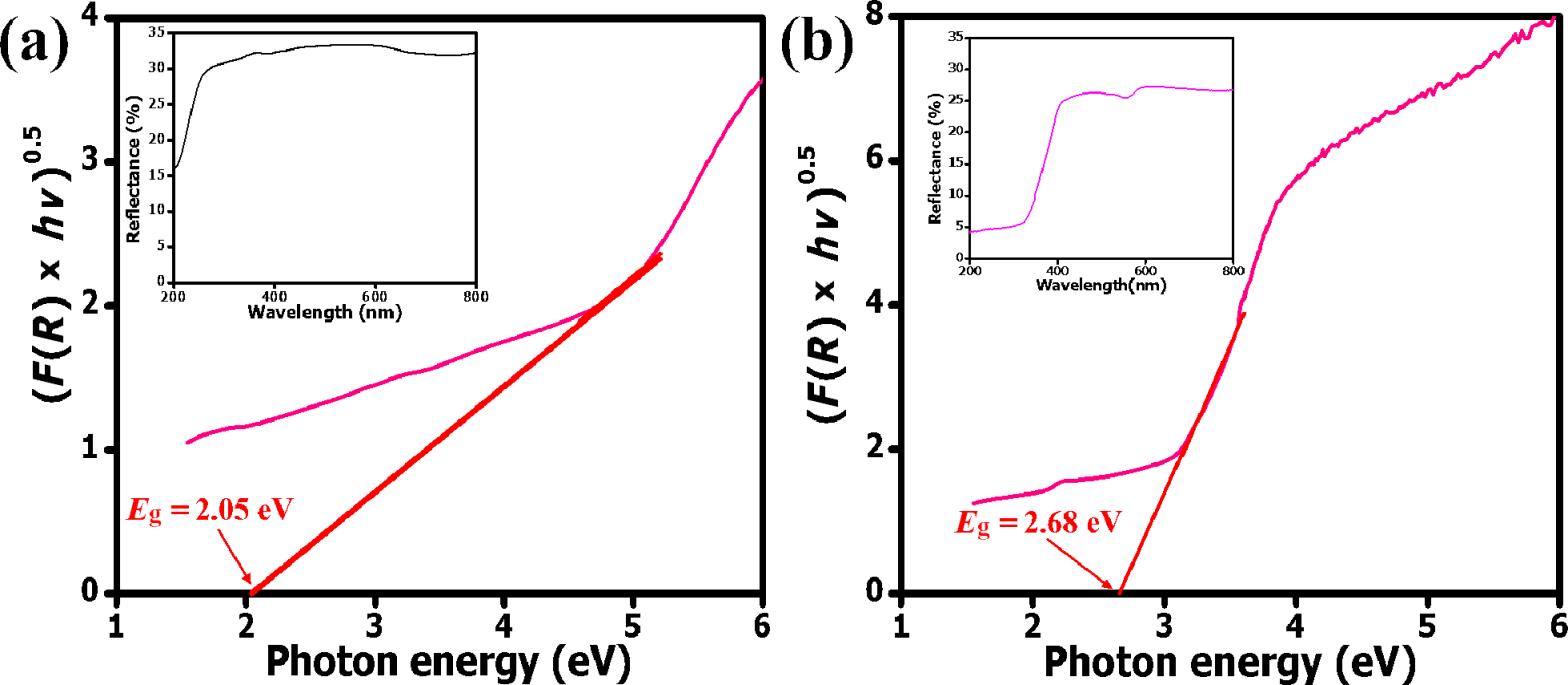
Indirect bandgap transition and diffuse reflectance UV–vis spectra (inset) of (a) mSiO_2_@NiPS and (b) mSiO_2_@NiPs/TiO_2_.

Bandgap energy and surface functionality of nanomaterials greatly influence their photoactivity. UV–vis diffuse reflectance spectroscopy is a useful technique for probing the optoelectronic properties, band structure and molecular energy levels of semiconductors. It gives relevant information on the optical activity of nanomaterials as it involves the excitation of photogenerated electrons and holes. The bandgap energy values of nanomaterials can be estimated by direct extrapolation of either the *F*(*R*) spectrum or from the maximum absorption wavelength [47,50]. The solid-state UV–visible reflectance spectra of mSiO_2_@NiPS and mSiO_2_@NiPS/TiO_2_ were recorded in the wavelength range of 200 to 800 nm and are presented in the insets of Figure 3. The bandgap energy (*E*_g_) values of the materials were obtained from the plot of the square root of the Kubelka–Munk function, (*F*(*R*)·*h*ν)^1/2^ as a function of the photon energy *h*ν. The bandgap energy of mSiO_2_@NiPS and mSiO_2_@NiPS/TiO_2_, as estimated by extrapolation of the intersection between the linear region of the *F*(*R*) curve and the *x*-axis, was 2.05 and 2.68 eV, respectively. The bandgap energy of mSiO_2_@NiPS was close to that reported in literature for nickel phyllosilicates [39].

Generally, interfacial interactions, material crystallinity, and the presence of dopants can readily tune the bandgap energy of core–shell nanomaterials [30,51]. The presence of TiO_2_ on the shell of the mSiO_2_@NiPS surface played a role in increasing the bandgap energy of the core–shell nanostructure. A similar shift in the bandgap energy value of TiO_2_ (3.2 eV) to approx. 2.7 eV for Ni^2+^-doped TiO_2_ was reported by Devi and co-workers [51]. They associated the shift in light absorption to the charge transfer between the interacting ions. Although there are only a few studies on nickel phyllosilicates, a prior investigation of their photoelectrochemical properties has shown that they are semiconducting materials with a possible photoactivity within the visible region [39]. Thus, nickel-containing compounds such as NiPS possess a narrow bandgap with absorption in the visible region. The incorporation of TiO_2_ could yield a material that absorbs light in the UV and visible region [29,52–53]. These results suggest that a change in the bandgap could readily reduce the recombination of electrons and holes during UV irradiation of mSiO_2_@NiPS/TiO_2_ and, consequently, improve its photocatalytic activity.

Infrared spectroscopy is a useful technique to determine the functional groups present in the silica and nickel phyllosilicate nanomaterials. Figure S2c in Supporting Information File 1 shows infrared absorption peaks of mSiO_2_ and mSiO_2_@NiPS at 813 and 1073 cm^−1^, characteristic of Si–O symmetric stretching and asymmetric stretching modes in silica and silicates [49,54]. It was difficult to specify the Si–O–Si stretching bands that are distinct for 1:1 nickel phyllosilicate, due to the broad band observed at 950–1100 cm^−1^, which also represents the band position for Si–O–Si in silica. In the wavenumber region of 950–1280 cm^−1^, the mSiO_2_ spheres exhibited a more intense band, suggesting a well-formed high-density SiO_2_ network [55]. A slight decrease in the intensity of the Si–O–Si bands was observed in mSiO_2_@NiPS. The mSiO_2_@NiPS/TiO_2_ bands recorded between 956 and 1220 cm^−1^ are indicative of Si–O–Si and Ti–O–Si stretching modes, revealing the presence of silica, silicates, and titania [56]. The intensity of these bands was smaller than that of mSiO_2_ and mSiO_2_@NiPS, indicating that the TiO_2_ particles had coated the mSiO_2_@NiPS surface. However, it was challenging to ascertain the peaks associated with hydroxy groups bonded to nickel atoms (δ(NiO–H) band) in NiPS as multiple peaks were recorded between 630 and 710 cm^−1^ [57].

### XPS analysis of mSiO_2_@NiPS and mSiO_2_@NiPS/TiO_2_

**Figure 4:**
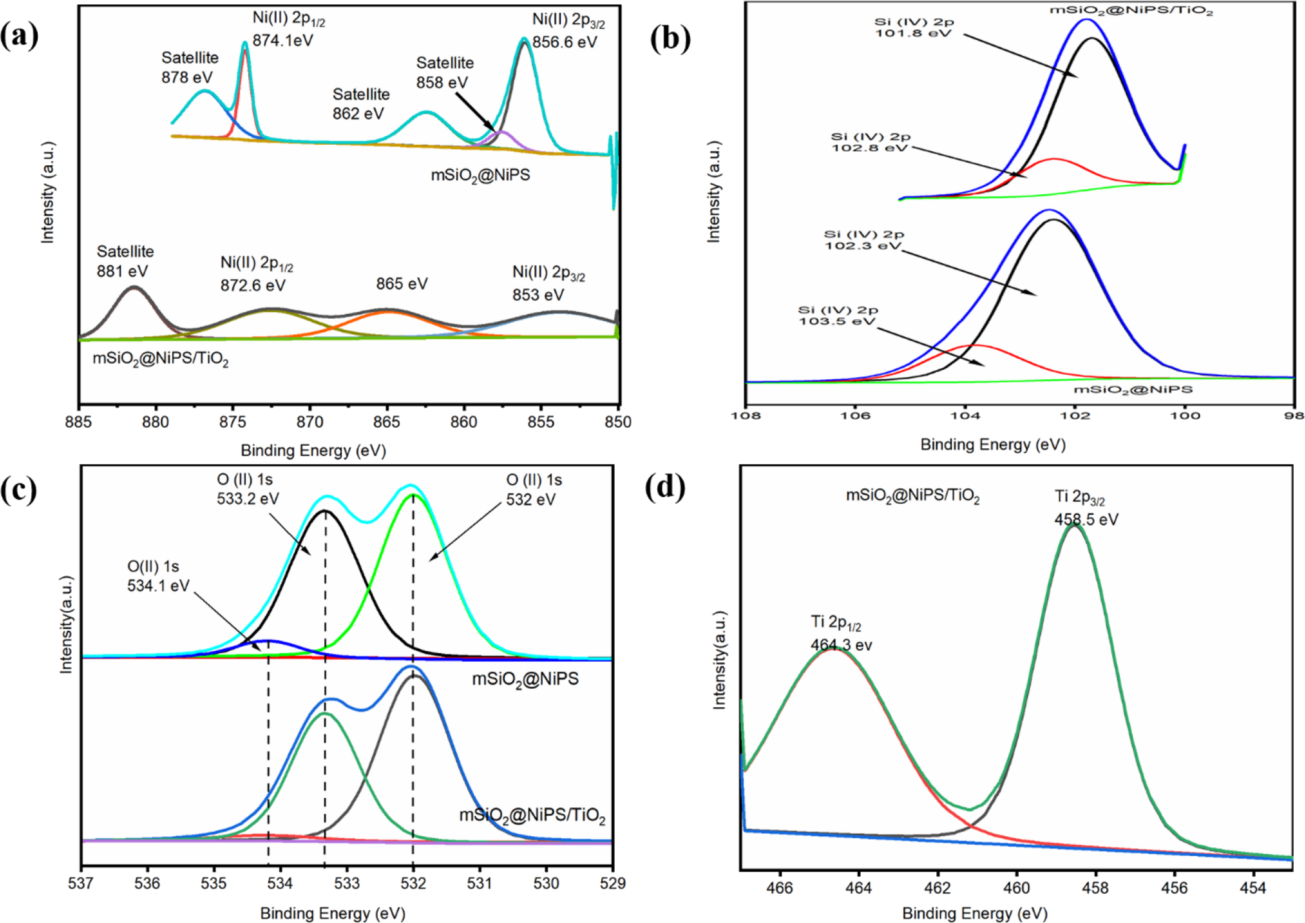
XPS spectrum of mSiO_2_@NiPS and mSiO_2_@NiPS/TiO_2_: (a) Ni 2p, (b) Si 2p, (c) O 1s, and (d) Ti 2p.

X-ray photoelectron spectroscopy (XPS) was used to study the surface composition of mSiO_2_@NiPS and mSiO_2_@NiPS/TiO_2_. The complete scans of the samples are shown in Figure 4, indicating the presence of Ni 2p, O 1s, Si 2p and Ti 2p. Figure S4 in Supporting Information File 1 presents the wide-scan survey spectra of mSiO_2_@NiPS and mSiO_2_@NiPS/TiO_2_, confirming the coating of TiO_2_ on the mSiO_2_@NiPS surface. For the mSiO_2_@NiPS catalyst, the Ni 2p_1/2_ and Ni 2p_3/2_ peaks (and their respective satellites) were readily detected at 874.1 and 856.6 eV, respectively (Figure 4a). The peak at 856.6 eV can be ascribed to the existence of 1:1 NiPS (formula: Ni_3_Si_2_O_5_(OH)_4_) [58–59]. This agrees with reports in the literature that describe lower Ni 2p_3/2_ binding energies for 1:1 phyllosilicates compared to that of 2:1 phyllosilicates [59–60]. Moreover, no peak originating from NiO (which has a lower binding energy at Ni 2p_3/2_ levels of less than 855 eV) was observed. This was confirmed by the appearance of the satellite splitting peaks with a separation between the Ni 2p_3/2_ primary line and the satellite of less than 6 eV, which is smaller than that of nickel oxides [60–61].

The data for the mSiO_2_@NiPS/TiO_2_ suggest a decrease in binding energies (Ni 2p_1/2_ at 872.6 eV and Ni 2p_3/2_ at 853 eV), possibly indicative of an interaction with TiO_2_ and the weakening of interactions between nickel and phyllosilicate [58]. However, a conclusive statement on the interaction between Ni and TiO_2_ is not possible. The XPS data in Supporting Information File 1, Table S1 shows that the non-normalised Ni and Si surface concentrations are 6.8 and 4.2 atom %, respectively, indicating a surface coverage of the SiO_2_ core by NiPS. The data is also consistent with a 1:1 (as opposed to a 2:1) NiPS structure. The addition of the small amount of TiO_2_ to mSiO_2_@NiPS resulted in a coverage of Ni and Si as shown by the drop in their surface concentrations (1.8 atom % for Ni and 1.9 atom % for Si).

The binding energy of the Si 2p peaks is 102.3 and 103.5 eV in mSiO_2_@NiPSs and 101.8 and 102.8 eV in mSiO_2_@NiPS/TiO_2_ (Figure 4b), suggesting the presence of both silica and silicates. The binding energy of Si 2p in SiO_2_ (approx. 103.5 eV) is higher than in silicates (102–103 eV) [60,62–64]. This illustrates that the samples were comprised of silicate and composites of nickel phyllosilicate with SiO_2_. The lower binding energies of the Si 2p peak in mSiO_2_@NiPS/TiO_2_ indicate a possible interaction between the silicates and TiO_2_ [65].

The O 1s spectrum was deconvoluted into three peaks with binding energies of 534.1, 533.2, and 532 eV (Figure 4c). The peaks with lower binding energy correspond to the presence of lattice oxygen bonded to Si, Ni, and/or Ti while the higher binding energy is due to defect oxygen associated with the presence of surface hydroxy groups [56]. Figure 4d shows two fitted peaks for Ti 2p for mSiO_2_@NiPS/TiO_2_ at binding energies of 458.5 and 464.3 eV, confirming the successful deposition of TiO_2_ on the mSiO_2_@NiPS matrix to form the mSiO_2_@NiPS/TiO_2_ composite. The two peaks correspond to the Ti 2p_3/2_ and Ti 2p_1/2_ states, respectively, and are consistent with the presence of Ti^4+^ surface states in Ni^2+^-doped TiO_2_ nanomaterials [66].

### Photocatalytic activity

**Figure 5:**
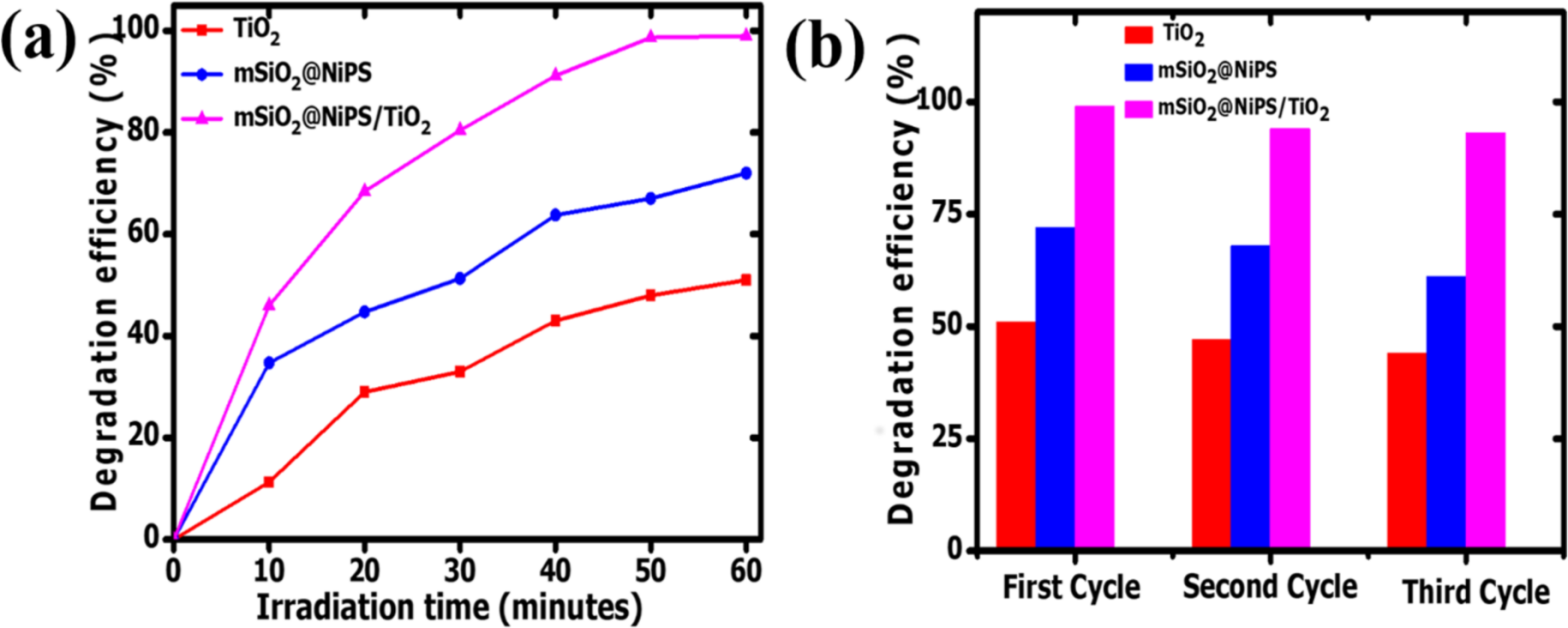
(a) The photocatalytic degradation of MV solution and (b) recyclability test for TiO_2_, mSiO_2_@NiPS, and mSiO_2_@NiPS/TiO_2_ nanostructures.

As a proof of concept, photocatalytic studies were carried out using the mSiO_2_@NiPS and mSiO_2_@NiPS/TiO_2_ nanostructures. TiO_2_, mSiO_2_@NiPS, and mSiO_2_@NiPS/TiO_2_ were tested for the degradation of methylene violet dye under UV light irradiation. Figure 5a shows the degradation results using the composites as well as that of pristine TiO_2_ for comparison. The degradation efficiency was found to increase with irradiation time for all the photocatalysts. The degradation efficiency was calculated to be 51, 72 and 99% for TiO_2_, mSiO_2_@NiPS, and mSiO_2_@NiPS/TiO_2_, respectively. The higher degradation efficacy measured for mSiO_2_@NiPS, compared to TiO_2_, can be ascribed to changes in surface area and to the unique mesoporous structure that allowed for improved dye adsorption. Both TiO_2_ and mSiO_2_@NiPS exhibited lower degradation efficacy than mSiO_2_@NiPS/TiO_2_, which can be associated with the possible occurrence of electron–hole recombination effects. The superior photoactivity of mSiO_2_@NiPS/TiO_2_ can be ascribed to a good interfacial interaction between NiPS and TiO_2_, which could inhibit electron–hole recombination, leading to faster charge transport and photodegradation.

Typically, the photocatalytic degradation efficacy of TiO_2_ depends on the surface area and metal-ion doping on the surface. Metal-ion doping of TiO_2_ influences its interfacial charge-transfer properties [51]. Differences in electronegativity and ionic radius between the metal ions and titania can alter the concentration of oxygen vacancies in the TiO_2_ lattice. Hence, the higher electronegativity of Ni^2+^ in NiPS [43] can induce defect sites within the structure and, consequently, alter light absorption and charge-transfer processes [51,67]. These oxygen vacancies easily act as hole traps that lower the charge-carrier recombination rate, resulting in more free electrons that can give rise to more superoxide radicals upon reaction with adsorbed surface oxygen [23].

Furthermore, the flake-like NiPS morphology could act as a suitable support for TiO_2_ creating a photocatalyst with improved photoactivity due to the presence of more active sites for the adsorption of the MV dye molecules. Additionally, from the diffuse reflectance UV–vis data, an optical bandgap of approximately 2.05 eV was obtained for mSiO_2_@NiPS while that of mSiO_2_@NiPS/TiO_2_ was higher with approx. 2.68 eV. Unlike other metal oxide binary systems where TiO_2_ is coated with SiO_2_ [68–69], in this study, the mSiO_2_@NiPS core–shell structure was coated with TiO_2_ nanoparticles, which readily absorb in the UV region. This suggests that the presence of TiO_2_ on the surface of mSiO_2_@NiPS could play a role in the absorption of light within the UV–visible region leading to an improved photoactivity.

Stability and reusability of the core–shell nanostructures as photocatalysts were studied. The measurements were repeated using the recycled catalysts for the degradation of the methyl violet solution. Figure 5b shows three cycles of degradation of the dye using the photocatalysts under UV light irradiation for 1 h. The degradation efficacy of the catalyst decreased with an increase in the number of cycles. This could be due to i) the loss of catalyst in the process of recovering the catalyst for repeated reactions or ii) the blocking of the active sites of the photocatalyst by photosensitive hydroxides on the photocatalyst surface. At the end of the third cycle, the values were 44, 61, and 93%, respectively. Therefore, the mSiO_2_@NiPS/TiO_2_ core–shell structure displayed good stability and reusability for the degradation of dye molecules and wastewater treatment.

**Figure 6:**
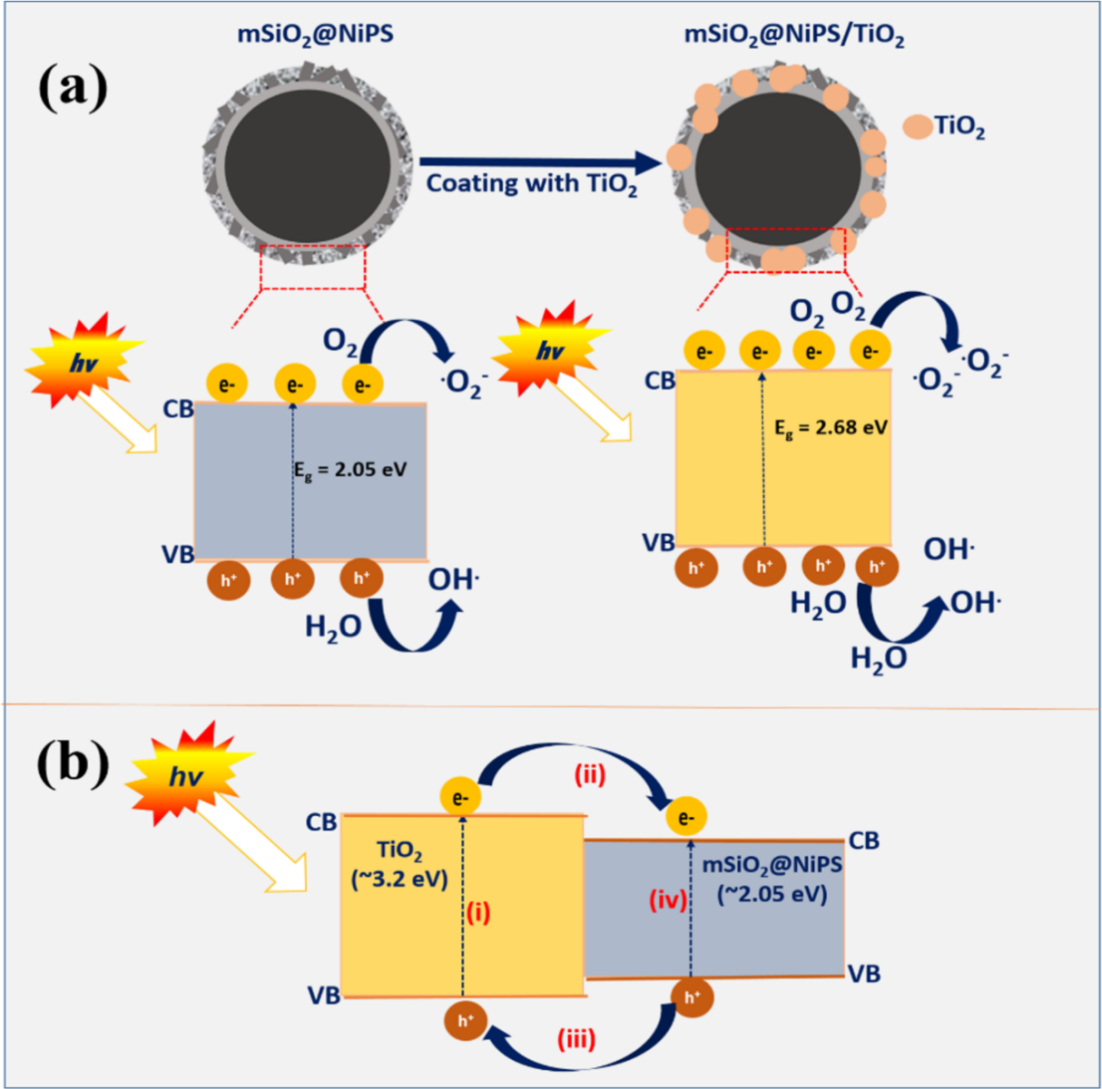
Proposed charge-transfer mechanism in mSiO_2_@NiPS and mSiO_2_@NiPS/TiO_2_ nanostructures under UV light illumination.

Figure 6 shows a possible mechanism for the photoactivity of mSiO_2_@NiPS and mSiO_2_@NiPS/TiO_2_. Upon light irradiation, electrons are excited from the valence band of both materials to the conduction band while holes are created in the valence band allowing for the separation of electrons and holes (Figure 6a). The electrons in the conduction band can react with oxygen to form reactive superoxide radicals, which oxidize the MV dye molecules [70]. Also, the holes in the valence band react with H_2_O to produce hydroxyl radicals that further degrade the dye. It is to be noted that no experiments were performed to examine the role of radicals in the reaction.

Figure 6b displays the possible charge-transfer process in the mSiO_2_@NiPS/TiO_2_ photocatalyst. The direction for the charge-transfer process in heterogeneous catalysts is determined by the positions of the conduction band (CB) and the valence band (VB) for each semiconductor. For example, nickel-containing compounds with Ni^2+^ ions are known to have a lower CB edge than TiO_2_ [30,71]. Therefore, in this study, it can be postulated that a photoinduced electron in the CB of TiO_2_ can be transferred to the CB of mSiO_2_@NiPS, whereby the latter acts as an electron trap (Figure 6b). The electron traps create more free holes that can form reactive hydroxyl radicals, which further degrade the MV dye [52]. The electron trapping process could also increase the lifetime of the holes and electrons [53]. As a consequence, the mSiO_2_@NiPS/TiO_2_ composite could degrade dye molecules more efficiently due to the enhanced charge-transfer process.

The core–shell structure also exhibited improved catalyst stability suggesting a good surface interaction between TiO_2_, SiO_2_, and NiPS. This agrees with previous reports on the catalyst stability of nickel–silica core–shell nanomaterials for the dehydrogenation of organic compounds and for methane reforming owing to the strong metal–support interaction [72–73]. Most importantly, the photocatalytic activity of the mSiO_2_@NiPS/TiO_2_ composite for MV degradation was superior to that of other related materials reported in the literature (Table 2) [18,74–80]. In addition, it was possible to achieve higher photodegradation efficacies at a lower catalyst dosage and within shorter irradiation times.

**Table 2:** Comparison of the photoactivity of the mSiO_2_@NiPS and mSiO_2_@NiPS/TiO_2_ core–shell nanostructure with other catalysts for the degradation of methyl violet dye.

Type of catalyst	Surface area (m^2^·g^−1^)	Catalyst dosage (g)	Irradiation time (min)	Degradation efficiency (%)	Ref.

SiO_2_/TiO_2_	99	0.005	180	71	[18]
Co–TiO_2_–SiO_2_	245	0.250	240	54	[74]
TiO_2_	66	0.025	240	32	[75]
Co-doped SBA15	690	1.000	150	61	[76]
ZnO	not stated	0.100	80	96	[77]
TiO_2_/calcium silicate	149	0.020	60	29	[78]
TiSiW_12_O_40_/TiO_2_	not stated	0.300	180	82	[79]
Sn@C-dots/TiO_2_	47	0.060	210	60	[80]
mSiO_2_@NiPS	165	0.050	60	72	this work
mSiO_2_@NiPS/TiO_2_	103	0.050	60	99	this work

## Conclusion

Mesoporous silica@NiPS and mesoporous silica@NiPS/titania core–shell nanostructures were successfully prepared by a simple deposition-precipitation method. The surface area of the mSiO_2_@NiPS/TiO_2_ was lower than that of the mSiO_2_@NiPS core–shell nanostructure indicating the blockage of some of the surface pores by TiO_2_. XPS analysis confirmed the formation of 1:1 nickel phyllosilicate as indicated by the binding energy values of the Ni 2p and Si 2p peaks. Among all the parameters in the experimental design, synergistic effects in mSiO_2_@NiPS/TiO_2_ played the most important role in influencing the photodegradation activity. Charge transfer between mSiO_2_@NiPS and TiO_2_ led to a faster charge-carrier separation and transportation at the interface, improving the photoactivity. The photocatalytic results showed that the mSiO_2_@NiPS/TiO_2_ composite exhibited excellent photocatalytic performance with a methyl violet degradation efficacy of 99% after 1 h, suggesting that the catalyst is appropriate for application in wastewater treatment and dye removal. Moreover, the mSiO_2_@NiPS core–shell nanostructure proved to be a suitable catalyst support for TiO_2_ nanoparticles creating a strong interfacial surface interaction between NiPS and TiO_2_, which resulted in good stability and reusability of the catalyst. The generation of a core–shell nanostructure with tunable surface and optoelectronic properties is a promising approach for the production of efficient photocatalysts.

## Experimental

### Starting materials

Ammonia solution (NH_4_OH, 25%; Fluka), ethanol (C_2_H_5_OH, 98%; Merck), tetraethylorthosilicate (TEOS, 98%; Aldrich), octadecyltrimethoxysilane (C_18_-TMS, 90%; Aldrich), urea (CH_4_N_2_O, 98%, Promark Chemicals), and nickel chloride hexahydrate (NiCl_2_·6H_2_O, 98%; Aldrich) were used without further purification. Anatase TiO_2_ nanoparticles were purchased from Sigma-Aldrich.

### Synthesis of mSiO_2_, mSiO_2_@NiPS and mSiO_2_@NiPS/TiO_2_ core–shell nanostructures

Ethanol (100 mL) was added to 10 mL of water and the mixture was stirred for 10 min. A solution of 10 mL of ammonia was added to the co-solvents and the resultant mixture was stirred for 20 min. TEOS (10 mL) was added to the solvent mixture, and the solution was stirred further at room temperature for 4 h. C_18_-TMS (2 mL) together with 5 mL of TEOS were thereafter added to the reaction mixture and the solution was stirred for 24 h [81]. The resulting product was centrifuged at 5000 rpm for 15 min. The obtained solid product was washed three times with a 50:50 mixture of ethanol and distilled water through centrifugation. The washed solid was dried overnight in an oven at 80 °C for 12 h followed by calcination at 500 °C for 6 h to form the solid SiO_2_@mesoporous SiO_2_ product, hereafter referred to as mSiO_2_.

Nickel phyllosilicate nanomaterials were formed on mSiO_2_ using a deposition-precipitation method. In a typical procedure, a 5 wt % loading of NiPS on mSiO_2_ spheres was obtained by dispersing a calculated amount of mSiO_2_ in 200 mL of distilled water, followed by the addition of nickel chloride hexahydrate (0.001 mol). The mixture was stirred in a round bottom flask placed in an oil bath and heated to 90 °C for 15 min. To this mixture, about (0.08 mol) of urea was added and the mixture was stirred for 5 h under reflux at 90 °C. The solution was cooled for 30 min and centrifuged at 5000 rpm for 15 min. The collected green solid was then dried at 100 °C for 12 h to give the mSiO_2_@NiPS core–shell nanostructure.

Finally, the mSiO_2_@NiPS/TiO_2_ composite was obtained by dissolving 0.15 g of mSiO_2_@NiPS and 0.15 g of anatase TiO_2_ in 20 mL of ethanol and stirring the resulting mixture for 1 h at room temperature. The solution was then centrifuged at 10000 rpm for 10 min and the resulting solid was dried at 80 °C for 12 h.

### Characterization

The morphology of the obtained samples was elucidated using transmission electron microscopy (FEI Technai G2 spirit electron microscope operating at 120 kV) and scanning electron microscopy (FEI Nova Nanolab 600 FIB/SEM). The N_2_ adsorption and desorption isotherms of the samples were recorded using a Micromeritics Tristar 3000 instrument at 77 K. Before running the experiment, the samples were degassed at 150 °C for 12 h in N_2_ gas. The surface area was determined by the BET method from N_2_ adsorption data and the pore size distribution was determined by the Barret–Joyner–Halenda (BJH) method. The diffuse reflectance (DR) UV–vis spectra were obtained in the range of 200–800 nm using a Cary 500 spectrophotometer equipped with a Praying Mantis diffuse reflectance accessory. The bandgap energy for the composite was calculated from the reflectance data (%*R*) by applying the Kubelka–Munk function (*F*(*R*)) [82]*.* The Kubelka–Munk function, *F*(*R*) was considered proportional to the absorbed radiation according to Equation 1 and Equation 2 [83]:

[1] $$F\left(R\right)=\frac{{\left(1-R\right)}^{2}}{2R}=\frac{\alpha }{S},$$

[2] $${\left(\alpha h\nu \right)}^{n}=\text{const}\left(h\nu -E_{\text{g}}\right),$$

where *F*(*R*) is the Kubelka–Munk function, α is the absorption coefficient and *S* is the scattering coefficient (*n* = 2 or 0.5 for direct or indirect transitions, respectively).

The functional groups on mSiO_2_@NiPS and mSiO_2_@NiPS/TiO_2_ composites were determined using a Bruker Tensor 27 Fourier-transform infrared (FTIR) spectrometer with measurements between 600 cm^−1^ and 4000 cm^−1^. The XPS analyses were obtained with a Kratos Axis supra spectrometer using an Al Kα source.

### Photocatalytic test

A glass reactor equipped with a 100 W high-pressure mercury lamp (Sol 2A, Newport 94022A model) was used and the temperature of the solution was maintained at 25 °C throughout the experiment. A methylene violet (MV) solution (10 ppm; 100 mL) was placed in the reactor followed by the addition of 0.05 g of the catalyst. The solution was magnetically stirred in the dark for 1 h to reach the adsorption–desorption equilibrium. The suspension was then irradiated under a UV light source under constant stirring. The photocatalytic reaction was monitored using UV–vis spectroscopy on each solution aliquot (1 mL) taken at 10 min intervals. The UV–vis spectra recorded at the maximum absorbance for methyl violet dye (464 nm) was used for evaluation of the photocatalytic properties. The photocatalytic degradation efficiency was estimated from the equation *D*% = (*C*_0_ − *C*/*C*_0_) × 100% (where *C*_0_ is the initial concentration and *C* is the concentration of MV solution after UV irradiation at a time *t*) [48,84]*.* After the catalyst was used for an hour, the remaining solution containing the dye was removed after centrifugation and the catalysts was washed three times with an ethanol/water mixture to regenerate it. The catalyst was then dried in an oven at 100 °C for 3 h and it was kept for reuse in the next cycle. This reused catalyst was added to a new MV solution in a 100 mL beaker and the mixture was stirred in the dark to equilibrate the adsorption–desorption mechanism. The rest of the procedure was repeated exactly as above for three cycles.

## Supporting Information

The Supporting Information contains additional information on the morphological features, Energy dispersive spectroscopy data, XRD patterns and specific surface area analysis of the core–shell nanomaterials.

File 1 Additional experimental results.
