# Supplementary material for: Unravelling the interfacial interaction in mesoporous SiO2@nickel phyllosilicate/TiO2 core–shell nanostructures for photocatalytic activity
Source: Beilstein J Nanotechnol. 2020 Dec 9;11:1834–46. doi: 10.3762/bjnano.11.165 (PMC7736695; doi:10.3762/bjnano.11.165)
Supplement: File 1 — Additional experimental results. [file Beilstein_J_Nanotechnol-11-1834-s001.pdf]

BEILSTEIN JOURNAL OF NANOTECHNOLOGY

Supporting Information

for

Unravelling the interfacial interaction in mesoporous SiO_2@nickel phyllosilicate/TiO_2 core–shell nanostructures for photocatalytic activity

Bridget K. Mutuma, Xiluva Mathebula, Isaac Nongwe, Bonakele P. Mtolo, Boitumelo J. Matsoso, Rudolph Erasmus, Zikhona Tetana and Neil J. Coville

Beilstein J. Nanotechnol. **2020,** *11,* 1834–1846. doi:10.3762/bjnano.11.165

Additional experimental results

License and Terms: This is a supporting information file under the terms of the Creative Commons Attribution License (https://creativecommons.org/licenses/by/4.0). Please note that the reuse, redistribution and reproduction in particular requires that the author(s) and source are credited and that individual graphics may be subject to special legal provisions.
The license is subject to the *Beilstein Journal of Nanotechnology* terms and conditions: (https://www.beilstein-journals.org/bjnano/terms)

Table S1: Summary of atom % concentration for the Si 2p, Si 2s, O 1s, Ni 2p, Ni 2s and Ti 2p spectra in the samples.

Sample name	Peak	Binding energy (eV)	concentration (atom %)
**$mSiO_2$@NiPS**	Si 2p	103	2.21
	Si 2s	154	2.08
	C 1s	285	69.24
	O 1s	532	19.53
	Ni 2s	977	6.72
	Ni 2p	856	0.21
**$mSiO_2$@NiPS/TiO_2**	Si 2p	102	0.85
	Si 2s	153	0.90
	C 1s	285	76.52
	O 1s	532	19.69
	Ni 2s	1000	1.93
	Ti 2p	459	0.10

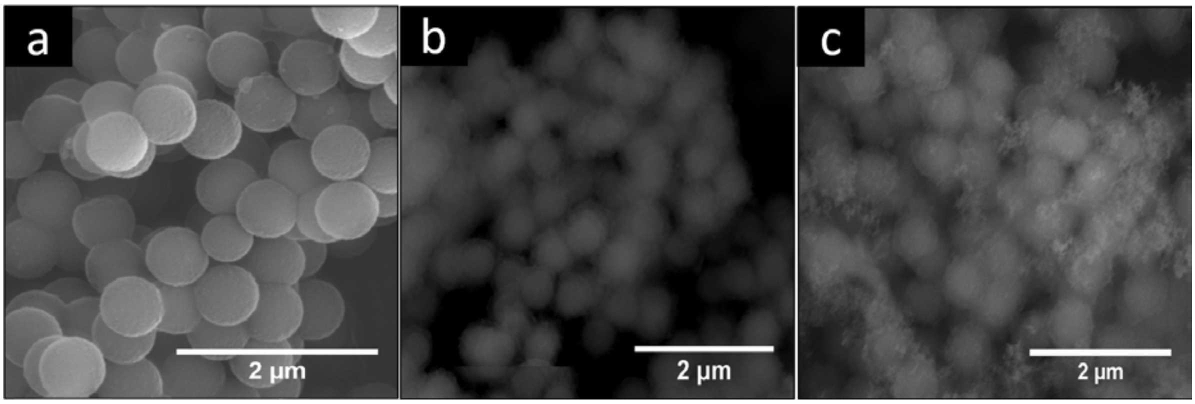

Figure S1: SEM images of (a) $mSiO_2$, (b) $mSiO_2$@NiPS and (c) $mSiO_2$@NiPS/TiO_2 core–shell nanomaterials.

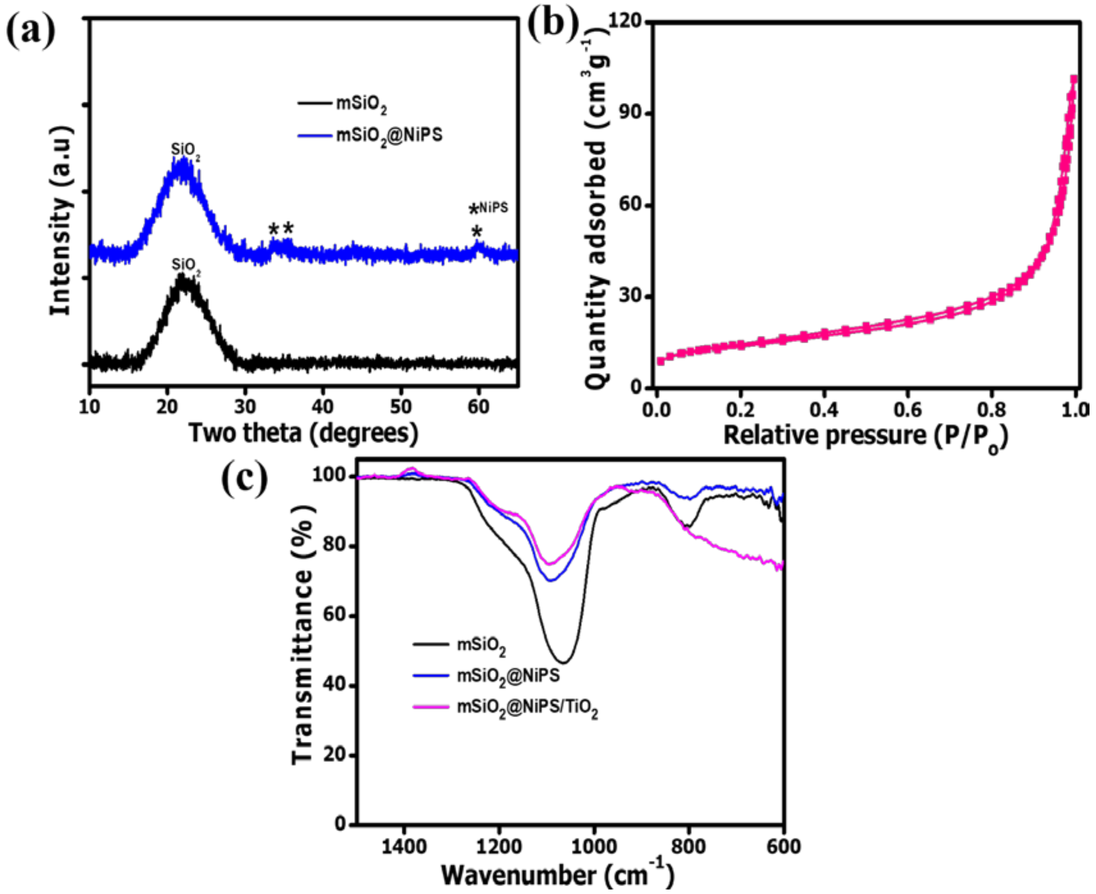

Figure S2: (a) XRD patterns $mSiO_2$ and $mSiO_2$@NiPS, (b) N_2 adsorption–desorption isotherm of TiO_2 and (c) FTIR spectra of $mSiO_2$, $mSiO_2$@NiPS and $mSiO_2$@NiPS/TiO_2.

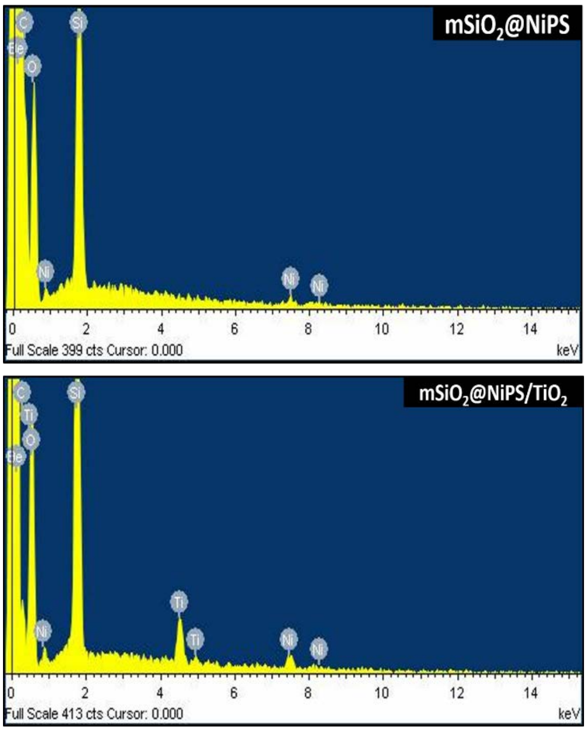

Figure S3: EDX spectra of the $mSiO_2$@NiPS and $mSiO_2$@NiPS/TiO_2 core–shell nanomaterials.

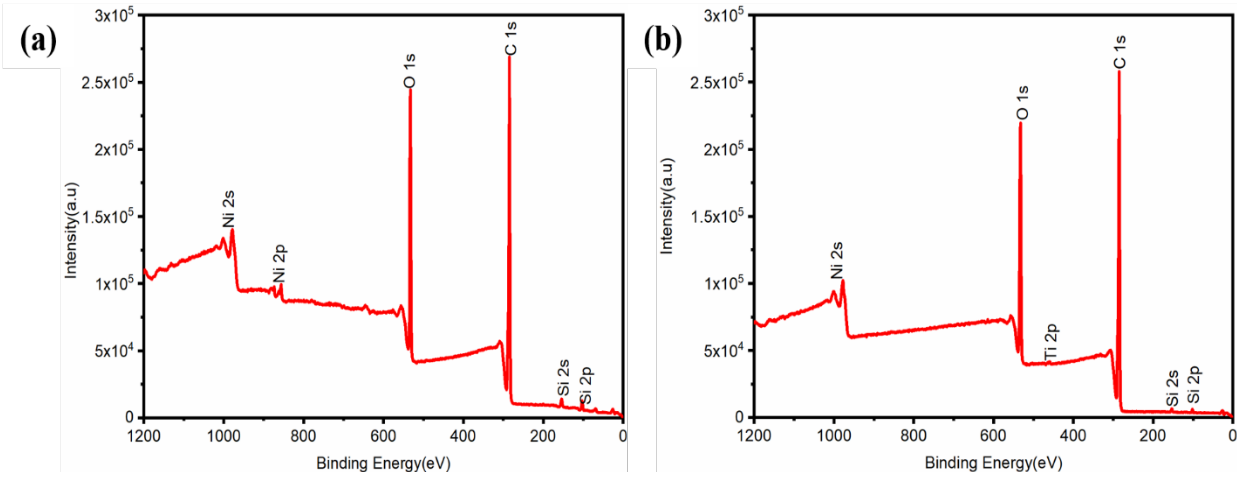

Figure S4: Wide-scan survey spectra of (a) $mSiO_2$@NiPS and (b) $mSiO_2$@NiPS/TiO_2 nanomaterials.
